# Targeted delivery of miR-34a-5p by phenylborate-coupled polyethylenimide nanocarriers for anti-KSHV treatment

**DOI:** 10.3389/fbioe.2023.1343956

**Published:** 2024-01-08

**Authors:** Fangling Li, Dongdong Cao, Lixia Yao, Wenyi Gu, Zhiyong Liu, Dongmei Li, Lin Cui

**Affiliations:** ^1^ School of Chemistry and Chemical Engineering, State Key Laboratory Incubation Base for Green Processing of Chemical Engineering, Shihezi University, Shihezi, Xinjiang, China; ^2^ School of Medicine, Shihezi University, Shihezi, Xinjiang, China; ^3^ Australian Institute for Bioengineering and Nanotechnology (AIBN), University of Queensland (UQ), Brisbane, QLD, Australia

**Keywords:** cationic copolymer nanocarriers, targeting function, nucleic acid delivery, anti-KSHV treatment, drug-carrying nanocomplex

## Abstract

Kaposi’s sarcoma-associated herpesvirus (KSHV) can infect a variety of cells and cause malignant tumors. At present, the use of microRNA (miRNA) for anti-KSHV is a promising treatment strategy, but the instability and non-specific uptake of miRNA still limit its use in the treatment of KSHV. In the present study, we constructed a nano-drug delivery system employing chemical grafting and electrostatic adsorption to solve the problems of easy degradation and low cell uptake of miRNA during direct administration. This nano-drug delivery system is to graft 4-carboxyphenylboric acid (PBA) and lauric acid (LA) onto polyethylenimine (PEI) through amidation reaction, and then prepare cationic copolymer nanocarriers (LA-PEI-PBA). The drug-carrying nanocomplex LA-PEI-PBA/miR-34a-5p was formed after further electrostatic adsorption of miR-34a-5p on the carrier and could protect miR-34a-5p from nuclease and serum degradation. Modification of the drug-carrying nanocomplex LA-PEI-PBA/miR-34a-5p by targeted molecule PBA showed effective uptake, increase in the level of miR-34a-5p, and inhibition of cell proliferation and migration in KSHV-infected cells. In addition, the drug-carrying nanocomplex could also significantly reduce the expression of KSHV lytic and latent genes, achieving the purpose of anti-KSHV treatment. In conclusion, these cationic copolymer nanocarriers with PBA targeting possess potential applications in nucleic acid delivery and anti-KSHV therapy.

## 1 Introduction

Kaposi’s sarcoma-associated herpesvirus (KSHV) is a double-stranded DNA virus that infects lymphocytes, endothelial cells, and nerve cells, and can cause Kaposi’s sarcoma (KS), primary effusive lymphoma (PEL), multicenter Castleman disease (MCD), and KSHV inflammatory cytokine syndrome (KICS) (Prieto-Barrios et al., 2018; Alzhanova et al., 2021; Naimo et al., 2021). After infecting cells, the virus mainly exists in a latent state, and only a limited number of viral genes are expressed in order to evade the immune response of host cells and maintain the survival of the virus (Li et al., 2018; Gam Ze Letova et al., 2021; Sharma and Zheng, 2021). When it is stimulated by hypoxia, phorbol ester induction, and a variety of inflammatory factors, it will quickly enter the lytic state and produce infectious virus particles, thus infecting neighboring cells (Long et al., 2021; Chinna et al., 2023; Davis et al., 2023).

At present, the involvement of miRNA from KSHV and host cells in the regulation of latent and lytic switches has received more and more attention (Alomari and Totonchy, 2020; Kumar et al., 2022). miRNA is a class of small endogenous non-coding RNAs that can simultaneously regulate multiple target genes and play a unique tumor suppressive role in tumor occurrence, development, and metastasis through inhibition or degradation of the RNA translation (Hussein et al., 2019; Liu et al., 2019; Ma et al., 2022). Wu et al. (2022) reported that low-expressed miR-34a-5p in KSHV-infected cells could regulate the level of c-Fos and target the 3′-UTR region, thereby inhibiting cell proliferation and the expression of KSHV-related genes; thus miR-34a-5p may be used as a nucleic acid drug for the treatment of KSHV infection. However, its poor stability and non-specificity *in vivo* environment lead to low cell uptake efficiency, thus limiting its further application (Wang et al., 2019). Therefore, finding effective delivery methods to enhance its stability may lead to better anti-KSHV therapeutic effects.

The rapid development of nanomedicine provides advantages such as stability, targeting, safety, and versatility for gene delivery (Li and Kataoka, 2021; Wen et al., 2023). The nucleic acid delivery system based on a non-viral vector has many advantages such as low immunogenicity, good biocompatibility, high loading efficiency, and abundant sources (Jiao et al., 2020; Jiang et al., 2022; Tong et al., 2023). These delivery carriers mainly include cationic polymers (Van Bruggen et al., 2019; Feng et al., 2022), micelles (Ivana et al., 2021; Winkeljann et al., 2023), liposomes (Obuobi et al., 2020; Lohchania et al., 2022), dendrimers (Choudhury et al., 2022; Liyanage et al., 2022), and nanoparticles (NPs) (Vaughan et al., 2020; Sharma et al., 2022; Hu et al., 2023). Among them, cationic polymers have been widely studied and have a wide variety of controllable structure designs, which can form nanocomplexes by electrostatic interaction containing negative nucleic acids (You et al., 2018), and then carry out *in vivo* and intracellular transport. Polyethylenimine (PEI) is often used as a cationic carrier for gene delivery, but unmodified PEI can be cytotoxic (Wang et al., 2016; Li et al., 2020).

Li et al. (2015) and Truong et al. (2013) reported that the introduction of hydrophobic components into cationic polymer carriers can improve the stability of carrier delivery genes, promote cell uptake, and activate endosome escape. Lauric acid, a natural fatty acid found in human sebum and coconut (Li et al., 2023), is hydrophobic at one end of the molecular chain and is introduced to PEI to decrease toxicity and improve endosome escape.

4-carboxyphenylboric acid (PBA) is a Lewis acid capable of forming a reversible borate structure with compounds containing cis-diol. Based on this, PBA can be used as a ligand that specifically binds to sialic acid (SA) receptors, targeting tumor sites (Zhao et al., 2016; Chen et al., 2020). The content of SA on the membrane of cancer cells is significantly higher than that of normal cells, which makes it a tumor marker. The diagnosis and treatment of tumors based on SA has important clinical value (Monteiro et al., 2020). Under weakly acidic conditions of tumor tissue, PBA and SA can form stable borates. Therefore, nanodrug delivery systems using PBA as targeted ligands have been widely developed for the targeted delivery of anti-tumor drugs (Liu et al., 2018). Li et al. (2023) have prepared an acid-responsive nanoassembly for tumor active targeting, which is used to eliminate nucleated *Fusobacterium* (Fn) in tumors to enhance cancer treatment. In the nanoassembly, PBA can specifically bind to the overexpressed SA site on the surface of cancer cells and promote the uptake of drugs through receptor-mediated endocytosis, significantly inhibiting tumor growth.

Our team has reported a folate-targeted cationic copolymer nanocarrier for the delivery of miR-34a-5p (Fangling et al., 2023). It is very meaningful for anti-KSHV therapy to develop more targeted nano-drug delivery carriers while protecting miR-34a-5p. We grafted lauric acid (LA) and small molecule targeting substance PBA onto PEI through a simple “one-pot method” to form cationic copolymer nanocapsular LA-PEI-PBA. The whole process utilized the amidation reaction between the carboxyl group on LA and PBA and the amino group on PEI. This not only reduces the toxicity of PEI but also provides the targeting function for the vector. LA-PEI-PBA is electrostatic adsorbed with miR-34a-5p to form the final LA-PEI-PBA/miR-34a-5p, which can effectively protect and deliver miR-34a-5p to play the role of anti-KSHV treatment. These findings help to provide some new ideas for anti-KSHV treatment.

## 2 Materials and methods

### 2.1 Materials

4-carboxyphenylboric acid (PBA), lauric acid (LA), and polyethylenimine (PEI, MW1800) were purchased from Shanghai Aladdin Biochemical Technology Company, 1-ethyl-(3-dimethylaminopropyl) carbodiimide hydrochloride (EDC) was purchased from Zhengzhou Alpha Biochemical Company, N-hydroxysuccinimide (NHS) was purchased from Shanghai McLean Company, and methanol was purchased from Tianjin Fuyu Fine Chemical Company.

### 2.2 Synthesis and characterization of carrier

#### 2.2.1 Preparation of LA-PEI-PBA

PBA (221.6 mg, 1.296 mmol), LA (135 mg, 0.66 mmol), EDC (391.3 mg, 2.0 mmol), and NHS (200 mg, 1.7 mmol) were added to methanol (20 mL) and stirred at room temperature for 2 h. Then PEI (200 mg, 0.33 mmol) was dissolved in 6 mL methanol and slowly added to the above mixture, and we continued to stir for 24 h. Then, the solution was transferred to a dialysis bag (MWCO 1000 Da), where it underwent dialysis with deionized water for 3 days. It was then frozen in the refrigerator and finally freeze-dried in the freeze dryer to obtain white solid LA-PEI-PBA.

#### 2.2.2 Structural characterization of the nanocarriers

The LA-PEI and LA-PEI-PBA were characterized by ^1^H NMR using a 400 MHz nuclear magnetic resonance spectrometer (Switzerland, AVANCE III HD). Ultraviolet-visible spectrophotometers (UV-8000S, Shanghai, Yuanxi) were used to test the products, and the spectral scanning range was 200–800 nm. The sample was determined using a Fourier transform infrared spectrometer (FTIR, VERTEX 70, Bruker, USA), which mixed the sample with KBr and ground it for testing after pressing the tablet; the determination range was 400–4000 cm^−1^ with a resolution of 4 cm^−1^.

### 2.3 Preparation and characterization of drug-carrying nanocomplex

#### 2.3.1 Preparation of LA-PEI-PBA/miR-34a-5p

LA-PEI-PBA was first prepared into an aqueous solution with a concentration of 1 mg/mL, which was evenly dissolved by 100 W ultrasound, and then the solution of LA-PEI-PBA and miR-34a-5p was mixed according to different mass ratios and swirled for 30 s. Finally, the drug-carrying nanocomplex LA-PEI-PBA/miR-34a-5p was obtained after standing for 30 min. LA-PEI without modified PBA and miR-34a-5p were prepared by the same method as described above, and the obtained LA-PEI/miR-34a-5p was used as the control group in the follow-up experiment.

#### 2.3.2 Gel electrophoresis retardation test

The binding ability of the nanocarriers to miR-34a-5p was tested by gel retardation experiment. LA-PEI and LA-PEI-PBA were mixed with miR-34a-5p (1 *μ*g) according to different mass ratios and then stood at room temperature for 30 min to obtain drug-carrying nanocomplex. Then, the drug-carrying nanocomplex was added into the sampling hole of the agar-gel prepared in advance, and the agar-gel was placed in the electrophoresis apparatus with TRIS-ethyl acetate EDTA electrophoresis buffer (TAE buffer) at 110 V for 25 min. Finally, the gel imaging system was used to take pictures of the gel and observe the band on the gel to analyze the binding ability of the carrier to miR-34a-5p. 

#### 2.3.3 Particle size, Zeta potential, and morphology observation

The particle size and potential of the drug-carrying nanocomplexes were measured using the nano-size Zeta potential analyzer (Nanoplus-3), and the solvent was ultra-pure water. The morphology of drug-carrying nanocomplexes was observed by transmission electron microscopy (TEM, HT7700, Japan).

### 2.4 Protective assay

In order to detect the protective effect of the carrier on miR-34a-5p (1 *μ*g/per well), free miR-34a-5p, LA-PEI/miR-34a-5p, and LA-PEI-PBA/miR-34a-5p were mixed with 50% FBS (Fangling et al., 2023) or RNase A solution (10 *μ*g/mL) at room temperature for 1 h, and then agarose gel electrophoresis was performed (the same conditions as in 2.5).

### 2.5 *In vitro* hemolysis and stability test

LA-PEI and LA-PEI-PBA were prepared with 1×PBS solution, and 0.1%Tritonx-100 was used as a positive control, whereas PBS was used as a negative control. Then, various sample solutions, 0.1% Tritonx-100 and PBS solution (500 *μ*L) were absorbed into 5% red blood cell suspension (500 *μ*L), incubated at 37°C at 100 rpm for 2 h, and centrifuged at 2000 rpm for 10 min. Then, 100 *μ*L of supernatant was absorbed and placed in a 96-well plate, and the absorbance (OD) was measured at 450 nm by a microreader. We used the following formula to calculate the hemolysis rate of the sample:

$$\text{Hemolysis rate }\left(\%\right)=\left[\left(\text{sample group }-\text{ negative control}\right)/\left(\text{positive control }-\text{ negative control}\right)\right]\times 100$$ (1)

The LA-PEI and LA-PEI-PBA with a concentration of 1 mg/mL and the drug-carrying nanocomplexes LA-PEI/miR-34a-5p and LA-PEI-PBA/miR-34a-5p were dissolved in 50% FBS, and then 100 *μ*L of the above solution was absorbed and added to the 96-well plate. The solution was placed in a constant temperature incubator at 37°C and taken out at different times to measure the OD value at 415 nm. OD values can be regarded as turbidity changes to evaluate the stability of the sample.

### 2.6 Cell culture and cytotoxicity test

KMM cells were KSHV-transformed rat primary mesenchymal precursor cells and donated by Hangzhou Normal University, cultured in 90% DMEM and 10% FBS containing 150 *μ*g/mL hygromycin (Solarbio) at 37°C, 5% CO_2_ incubator. SK-RG cells were KSHV-infected SH-SY5Y cells described in our previous study (Cao et al., 2021), and cultured in a complete medium with 6 *μ*g/mL puromycin (Gibco) at 37°C, 5% CO_2_ incubator. The cytotoxicity of LA-PEI and LA-PEI-PBA were detected by MTT assay. SK-RG and KMM cells were placed in 96-well plates at a density of 5000 cells/well for 24 h. The supernatant was replaced with a complete culture medium without serum and then added into different concentrations of nanocarriers (5, 10, 20, 40, 60, 80, and 100 *μ*g/mL). After 48 h, 20 *µ*L of 3-(4,5-dimethylthiazol-2-yl)-2,5-diphenyltetrazolium bromide (MTT, Solarbio) was added to each well, and then cultured at 37°C in the dark for 4 h. The MTT medium was removed, and 100 *µ*L of dimethyl sulfoxide (Solarbio) was added. Finally, we measured the OD value at 490 nm (Bio-RAD).

### 2.7 Cell uptake assay

Immunofluorescence microscopy was used to observe the uptake of drug-carrying nanocomplexes in KMM cells. KMM cells were inoculated with a density of 1.0 × 10^5^ cells/well into a 6-well plate containing slides (climbing plates), cultured for 24 h, added with drug-loaded nanocomplex with red fluorescent miR-34a-5p-Cy3 (2 *μ*g) for 4 h, centrifuged with 1×PBS three times, and fixed with 4% paraformaldehyde for 30 min. We then removed the supernatant and added it to 1×PBS centrifugally three times to remove the residual 4% paraformaldehyde. Then, we dyed it with 1 *μ*g/mL DAPI solution for 10 min, removed the sliver, and placed it on a cover slide with 10 *µ*L anti-fluorescence quench agent. We gently sealed the surrounding area with neutral gum and observed the solution using a fluorescence microscope.

### 2.8 Cell proliferation assay

The KMM and SK-RG cells were incubated with the free miR-34a-5p, LA-PEI/miR-34a-5p, and LA-PEI-PBA/miR-34a-5p for 24 h. After adding 20 *μ*L MTT solution to every peer on days 1, 2, 3, 4, and 5, the mixtures were then cultured for another 4 h at 37°C with 5% CO_2_. The absorbance value at 490 nm was detected by an enzyme-labeled instrument.

### 2.9 Cell migration assay

After culturing with free miR-34a-5p, LA-PEI/miR-34a-5p, and LA-PEI-PBA/miR-34a-5p for 4 h, KMM and SK-RG cells were digested and centrifuged. Then, 1 mL of complete medium was added for re-suspension, mixed, and counted. We added 150 *μ*L cell suspension (containing 2 × 10^5^ cells) to the upper chamber and 800 *μ*L culture medium containing 10% FBS to the lower chamber and cultured for 36 h. The supernatant was removed, and 800 *μ*L 4% paraformaldehyde was used to fix the cells at room temperature for 30 min. Then, 1% crystal violet was dyed for 30 min and left to dry naturally. Five to six fields were randomly selected under the microscope for photo recording.

### 2.10 RNA extraction and real-time PCR

Total RNA of SK-RG and KMM cells was extracted using TRIzol reagent (Invitrogen). cDNA synthesis was used by RevertAid First Strand cDNA Synthesis kit (Thermo Fisher). Real-time PCR was used to detect cDNA recovered (SYBR Green PCR Kit; Qiagen). The primers are shown in Supplementary Table S1. Then real-time PCR was used to amplify the target fragment and the reaction conditions described in our previous study (Fangling et al., 2023).

### 2.11 Statistical analysis

The experimental data were processed and analyzed using Graph Pad Prism 9.0 and Origin 2018 software. The experimental data were represented as mean ± standard deviation. Multivariate analysis of variance was used to determine the statistical significance among all groups. Significant differences were expressed as follows: *p < 0.05, **p < 0.01, ***p < 0.001, and ****p < 0.0001.

## 3 Results and discussion

### 3.1 Synthesis and characterization of LA-PEI-PBA

**FIGURE 1:**
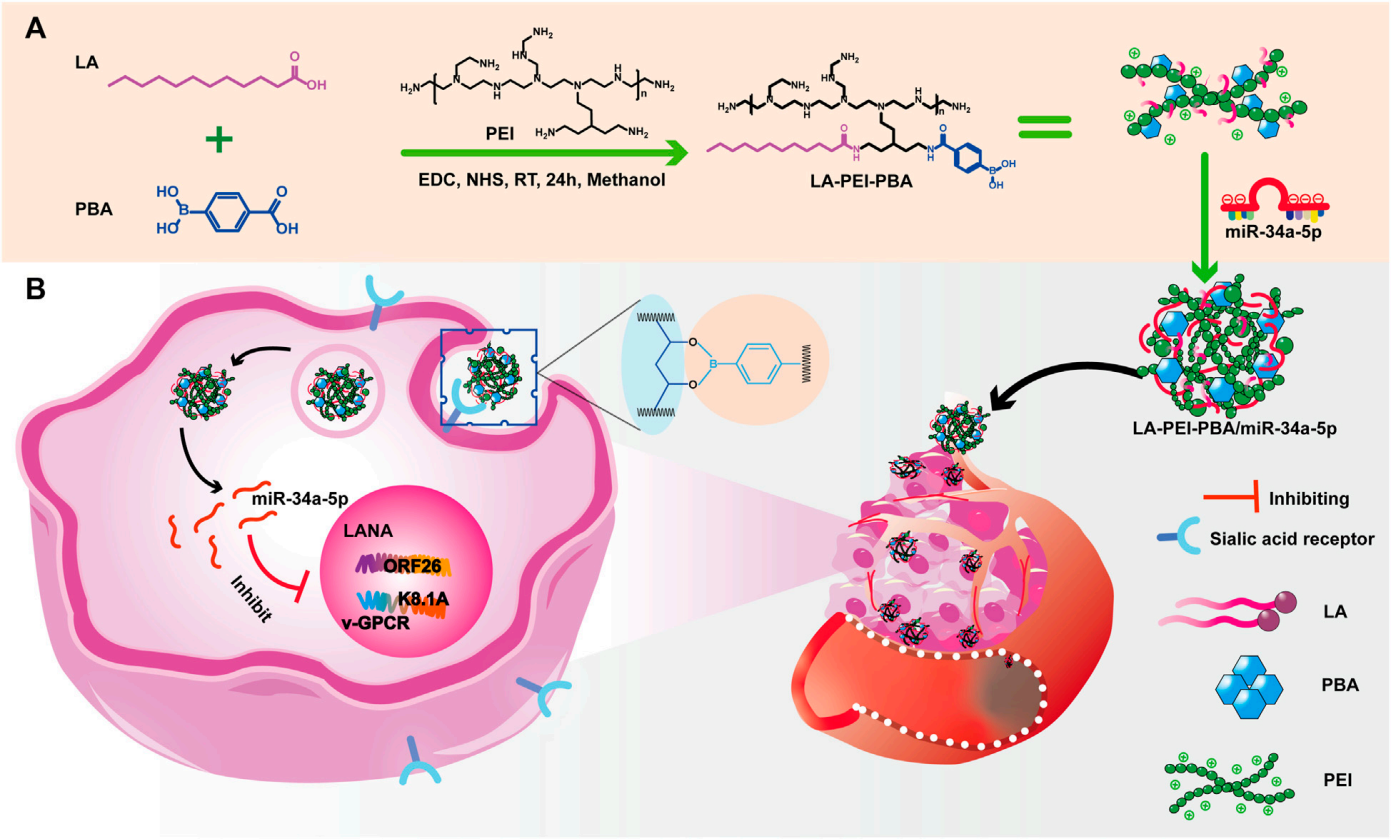
**(A)** LA-PEI-PBA synthesis process, **(B)** LA-PEI-PBA/miR-34a-5p anti-KSHV treatment diagram.

**FIGURE 2:**
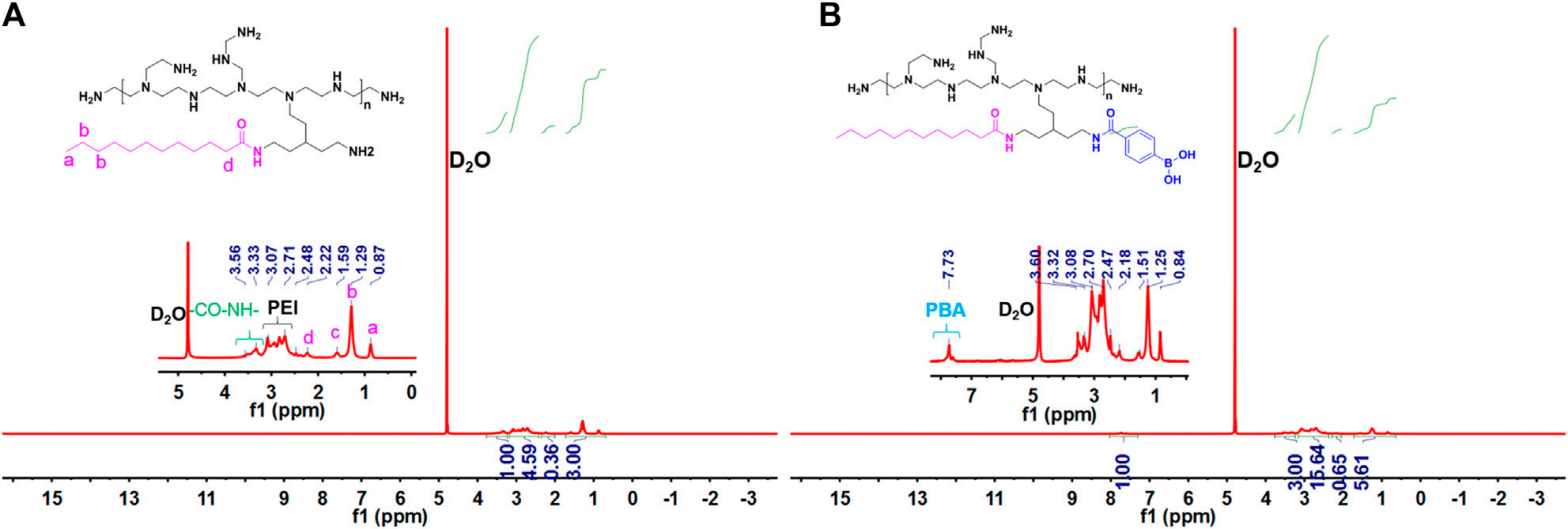
Structural characterization of LA-PEI-PBA. **(A)** LA-PEI and **(B)** LA-PEI-PBA ^1^HNMR.

The main synthesis steps of LA-PEI and LA-PEI-PBA are shown in Figure 1. LA was activated by the carboxyl group through EDC and NHS, formed an amide bond with PEI, and grafted to PEI, and LA-PEI was synthesized. The carboxyl groups on LA and PBA were activated by EDC and NHS at the same time, formed an amide bond with PEI, and grafted to PEI to prepare LA-PEI-PBA. The structures of LA-PEI and LA-PEI-PBA were characterized by ^1^H NMR. As can be seen from Figure 2A, in the control group, LA-PEI without PBA coupling had the characteristic peak of amide bond at 3.56–3.33 ppm, the characteristic peak of PEI at 3.07–2.48 ppm, and the characteristic peak of LA at 2.22 ppm and 1.59–0.87 ppm. This indicated that LA-PEI was successfully prepared. Then, LA-PEI-PBA was characterized by ^1^H NMR. Figure 2B shows that the characteristic peak of the benzene ring on PBA appeared at 7.73 ppm, and the chemical shift of the characteristic peak in the fat region was basically the same as that of LA-PEI, indicating that PBA had been successfully coupled to LA-PEI to prepare LA-PEI-PBA.

**FIGURE 3:**
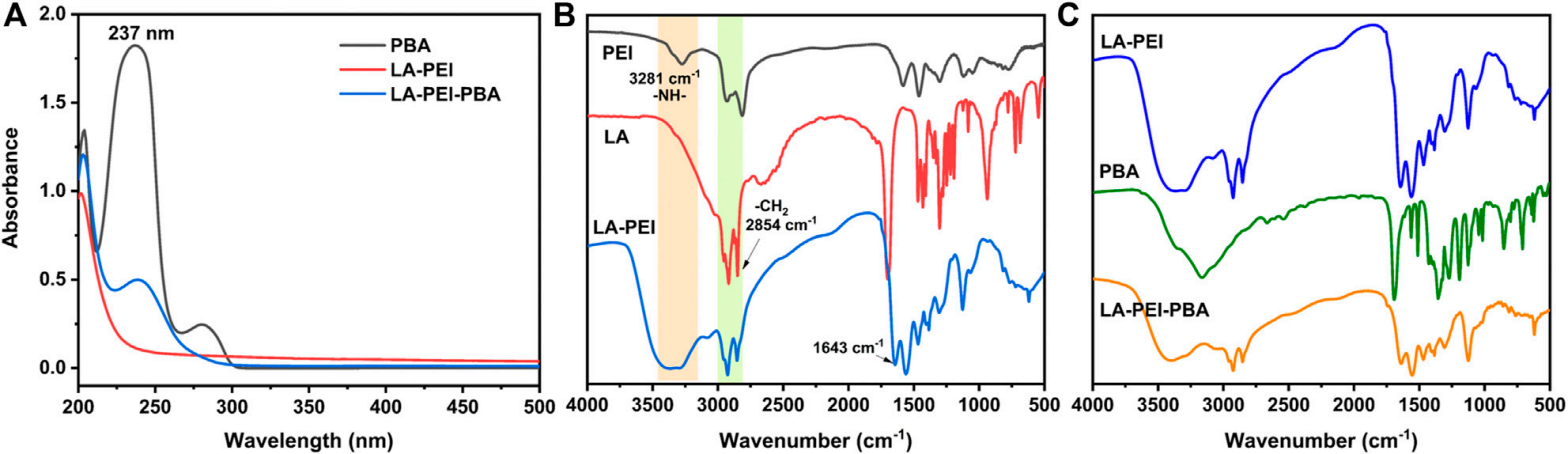
UV-Vis and IR absorption spectra of the carrier. **(A)** Ultraviolet-visible spectrum of LA-PEI-PBA, **(B)** infrared absorption spectrum of LA-PEI, and **(C)** infrared absorption spectrum of LA-PEI-PBA.

To further prove that the LA-PEI-PBA was successfully prepared, PBA, LA-PEI, and LA-PEI-PBA were tested by UV-Vis spectroscopy. As can be seen in Figure 3A, PBA’s characteristic absorption peak was 237 nm, but LA-PEI had no absorption peak at 237 nm. The characteristic absorption peak of PBA appeared at 237 nm in LA-PEI-PBA, which proves that PBA is coupled to LA-PEI. The infrared absorption spectrum of LA-PEI was composed of the -NH-stretching vibration peak of PEI at 3281 cm^−1^ and the -CH_2_ stretching vibration peak of LA at 2854 cm^−1^, and the characteristic absorption peak of the amide bond appeared at 1643 cm^−1^, as seen in Figure 3B, which proved that LA-PEI was prepared. Figure 3C shows that the infrared absorption spectrum of LA-PEI-PBA was composed of the absorption peak of LA-PEI, but the characteristic absorption peak of PBA does not appear, possibly because the grafting amount of PBA is too small, resulting in the absence of the infrared characteristic absorption peak. However, it could be proved by ^1^H NMR and UV-Vis that LA-PEI-PBA was successfully synthesized.

### 3.2 Preparation and characterization of LA-PEI-PBA/miR-34a-5p

**FIGURE 4:**
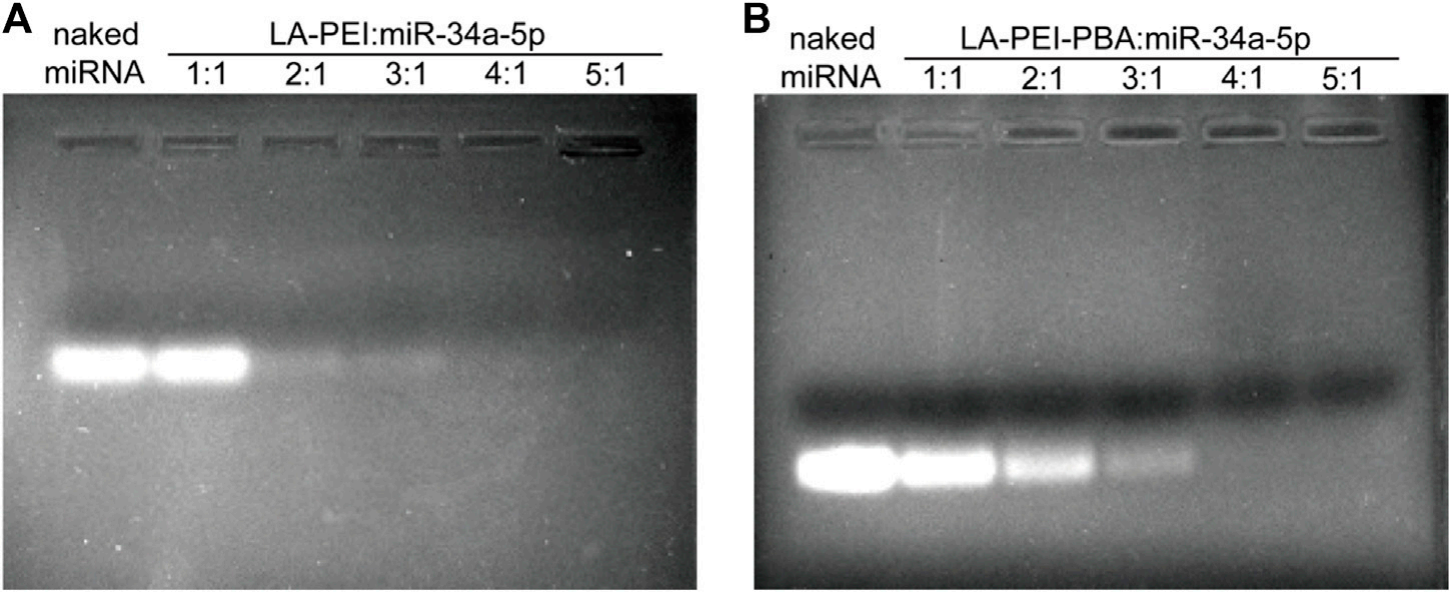
Gel electrophoresis of miR-34a-5p adsorption by carrier. **(A)** Gel electrophoresis of LA-PEI and miR-34a-5p; **(B)** Gel electrophoresis of LA-PEI-PBA and miR-34a-5p.

LA-PEI and LA-PEI-PBA formed stable drug-carrying nanocomplexes by electrostatic adsorption of miR-34a-5p. The adsorption and inclusion capacity of miR-34a-5p were evaluated by gel electrophoresis. If the vector could not effectively adsorb miR-34a-5p, bands of free miR-34a-5p would appear in the swim lane with the action of the current; otherwise, bands of miR-34a-5p would not appear. As shown in Figures 4A, B, when the mass ratio was ≥4, LA-PEI and LA-PEI-PBA could effectively contain miR-34a-5p and bind to form stable drug-carrying nanocomplexes, and PBA modification did not affect the adsorption capacity of the vector to miR-34a-5p.

**FIGURE 5:**
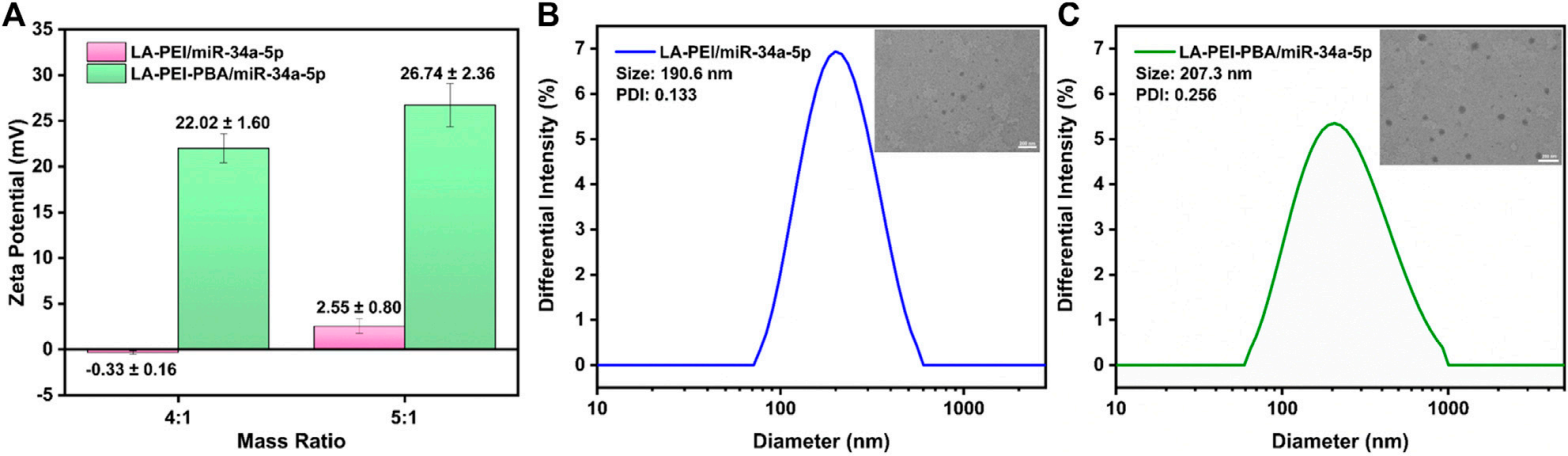
Potential and particle size of drug-carrying nanocomplex. **(A)** Potential of LA-PEI/miR-34a-5p and LA-PEI -PBA/miR-34a-5p. Data are presented as mean ± SD (*n* = 3), **(B)** particle size distribution and TEM image of LA-PEI/miR-34a-5p at a mass ratio of 5:1 (miR-34a-5p: 2 *μ*g), **(C)** Particle size distribution and TEM image of LA-PEI-PBA/miR-34a-5p at a mass ratio of 5:1 (miR-34a-5p: 2 *μ*g).

Next, we tested the potential values of LA-PEI/miR-34a-5p and LA-PEI-PBA/miR-34a-5p under the mass ratio of 4:1 and 5:1, respectively. With the increase of the mass ratio, the potential of LA-PEI/miR-34a-5p changed from −0.33 ± 0.16 mV to 2.55 ± 0.80 mV, indicating that LA-PEI could not fully contain miR-34a-5p when the mass ratio was 4:1, but at 5:1, the potential became positive, and LA-PEI could completely contain miR-34a-5p, as seen in Figure 5A. The reason is that miR-34a-5p is a negative nucleic acid, and when it is electrostatic adsorbed with the carrier, the negative potential becomes positive potential. At the same time, with the increase of the mass ratio, the potential of LA-PEI-PBA/miR-34a-5p changed from 22.02 ± 1.60 mV to 26.74 ± 2.36 mV, indicating that at the mass ratio of 4:1, LA-PEI-PBA could fully contain miR-34a-5p, and LA-PEI-PBA had a higher binding ability to miR-34a-5p. Figures 5B, C show that, at the best mass ratio of 5:1, the particle sizes of LA-PEI/miR-34a-5p and LA-PEI-PBA/miR-34a-5p were 190.6 nm and 207.3 nm, respectively. It could be seen from TEM images that the morphology of LA-PEI -PBA/miR-34a-5p was similar to a circle.

### 3.3 Protective effect of carriers on miR-34a-5p

**FIGURE 6:**
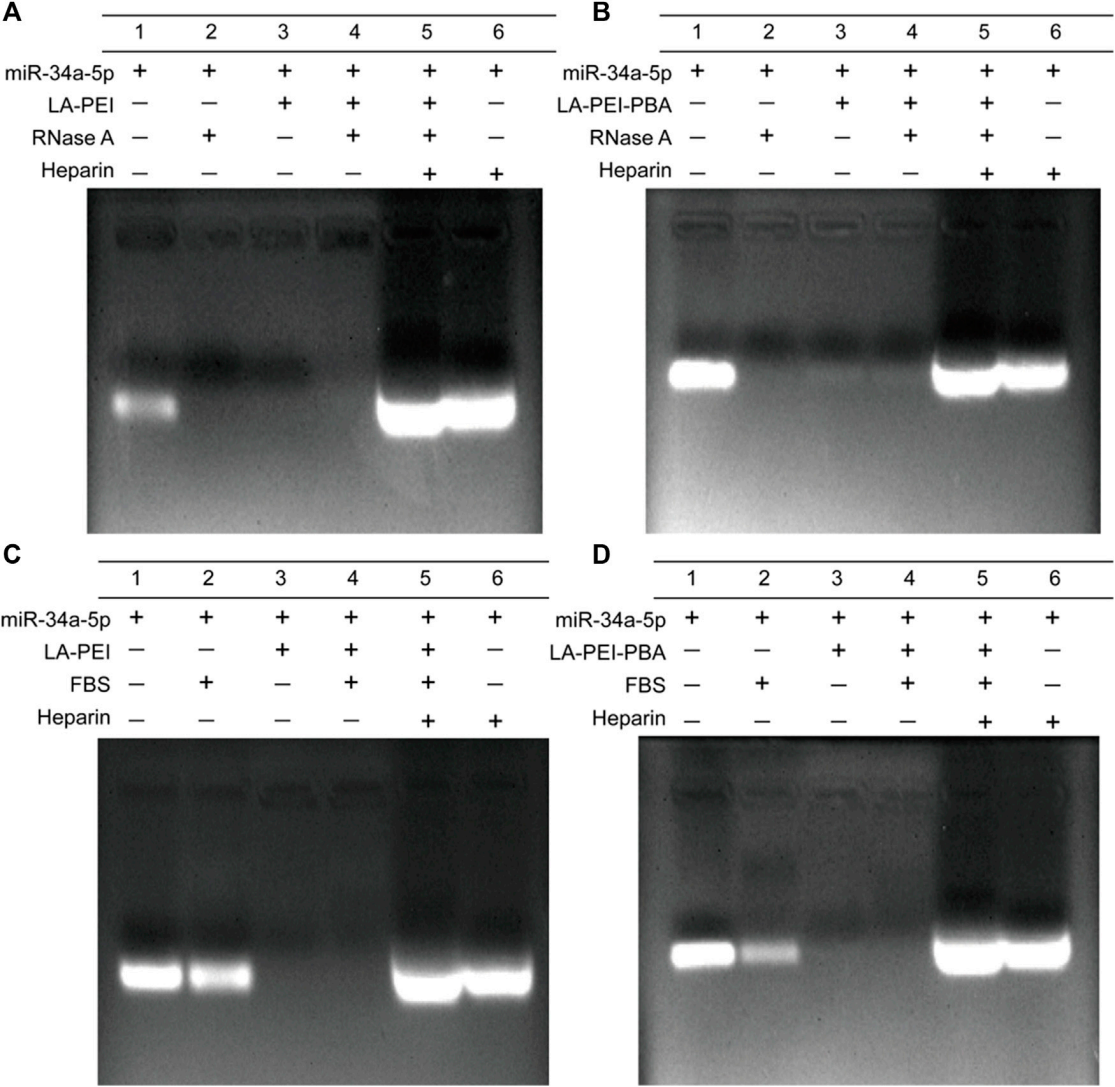
The protective effect of the carrier against miR-34a-5p was detected by gel electrophoresis. **(A, B)** 0.05 mg/mL of RNase A for 1 h, **(C, D)** 50% of FBS for 1 h (The mass ratio of carrier to miR-34a-5p was 5:1, and the miR-34a-5p was 1 *μ*g).

Free miR-34a-5p is easily degraded and unstable *in vivo*. We used gel electrophoresis to detect the protective effects of LA-PEI and LA-PEI-PBA on miR-34a-5p. As shown in Figures 6A, B, the free miR-34a-5p showed bright white bands in the swim lane, which disappeared after treatment by RNase A, indicating that RNase A completely degraded the free miR-34a-5p. However, LA-PEI/miR-34a-5p and LA-PEI-PBA/miR-34a-5p were treated with RNase A, and no bands appeared in the lane. When Heparin was added, which could compete with miR-34a-5p, bands of miR-34a-5p appeared again in the lane. This indicated that LA-PEI and LA-PEI-PBA could protect miR-34a-5p from the degradation of RNase A. Figures 6C, D also show the same results, except that FBS has a weak degradation effect on miR-34a-5p, and the band brightness of miR-34a-5p is weakened. All the above mentioned results indicate that LA-PEI and LA-PEI-PBA can effectively protect miR-34a-5p from the degradation of RNase A and FBS, thus improving the stability of miR-34a-5p.

### 3.4 Biosafety analysis of carriers *in vitro*

**FIGURE 7:**
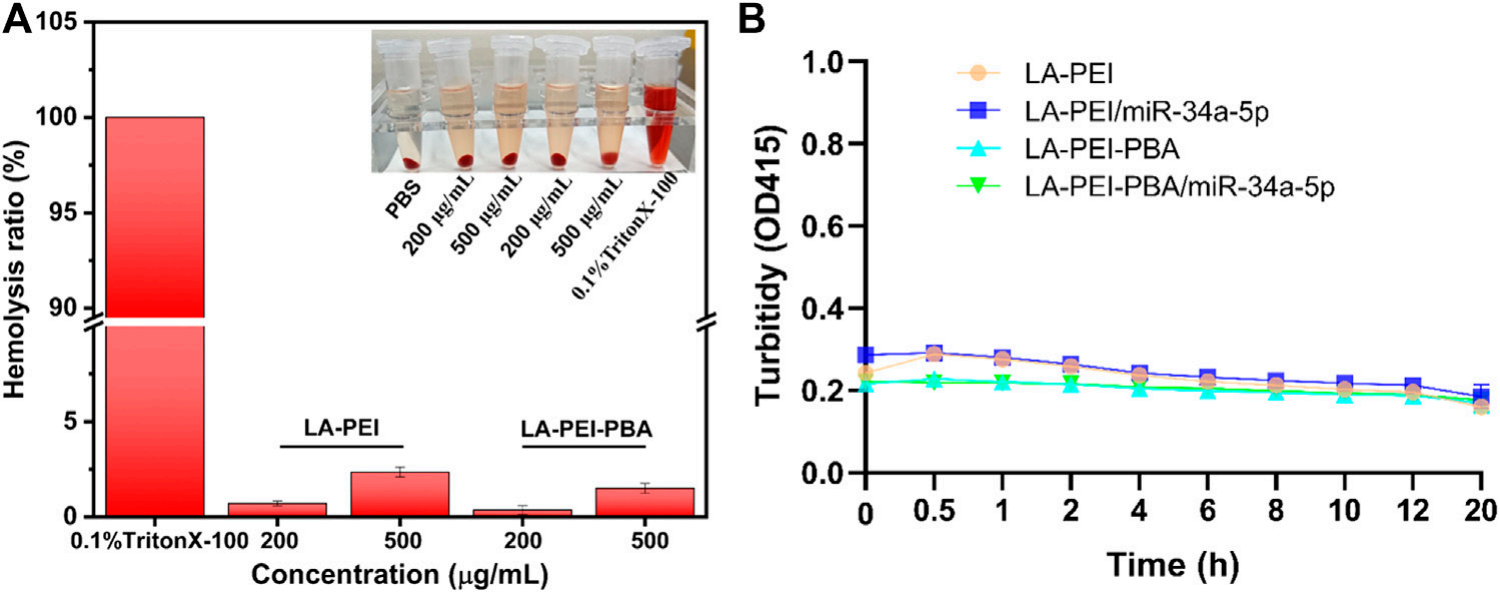
Hemolysis rate and stability analysis. **(A)** the hemolysis rates of LA-PEI and LA-PEI-PBA, **(B)** the OD values of LA-PEI, LA-PEI -PBA, LA-PEI/miR-34a-5p and LA-PEI -PBA/miR-34a-5p in 50% FBS. Data are presented as mean ± SD (*n* = 3).

The hemolysis effect of carriers on red blood cells is an important index to evaluate their biocompatibility. In Figure 7A, it can be seen that the color is bright red in the 0.1% TritonX-100 group, which means complete hemolysis. In contrast, the supernatant color of the LA-PEI and LA-PEI-PBA groups showed a light yellow color similar to the PBS group, and the erythrocyte was deposited at the bottom. When the carrier concentration was 200 *μ*g/mL and 500 *μ*g/mL, the hemolysis rates of LA-PEI to red blood cells were 0.70% and 2.35%, respectively, and the hemolysis rates of LA-PEI-PBA were 0.37% and 1.51% respectively, calculated according to Eq. (1). The hemolysis rates of LA-PEI and LA-PEI-PBA were both within the range of hemolysis safety standards (<5%), among which the hemolysis rate of LA-PEI-PBA on red blood cells was lower than that of LA-PEI, indicating that LA-PEI-PBA had better blood compatibility than LA-PEI.

PEI is a cationic polymer, often used as a carrier to deliver gene or nucleic acid drugs, but it is easy to bind to serum proteins in the blood, resulting in cytotoxicity and biosafety risks. Therefore, we tested the stability of LA-PEI, LA-PEI-PBA, and LA-PEI/miR-34a-5p and LA-PEI-PBA/miR-34a-5p in serum by measuring their absorbance changes (turbidity changes). Figure 7B shows that in 50% FBS, the absorbance of the carrier and drug-carrying nanocomplex did not change significantly within 20 h. Those results indicated that the carrier and drug-carrying nanocomplex were stable and did not bind to serum proteins to cause cytotoxicity.

**FIGURE 8:**
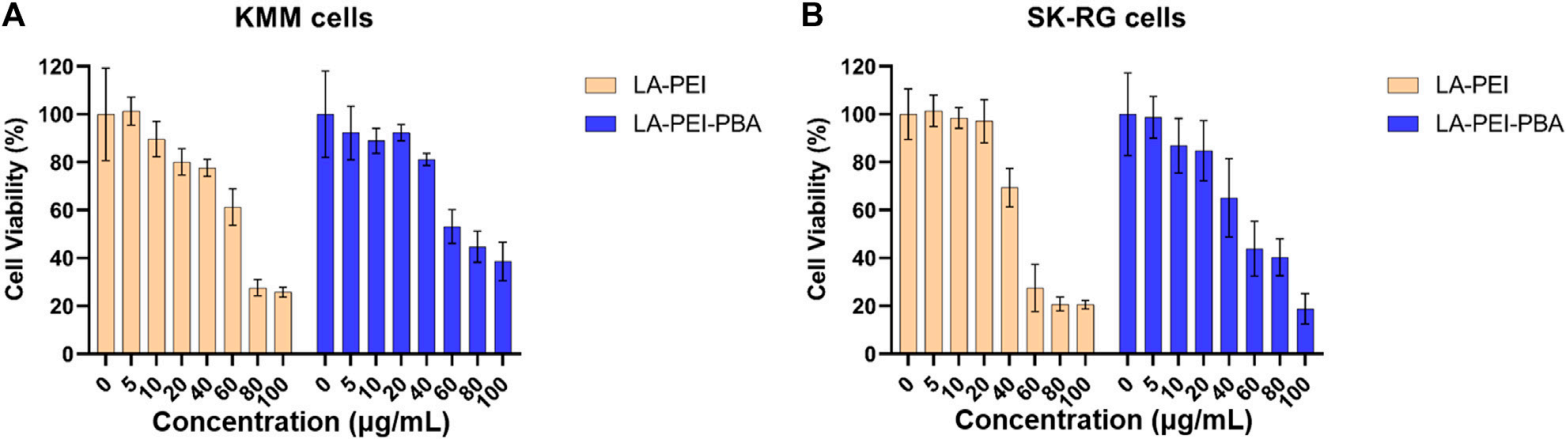
Cytotoxicity of nanocarriers. The cell survival rate of LA-PEI and LA-PEI-PBA with different concentrations after incubation with KMM **(A)** and SK-RG **(B)** cells for 48 h. Data are presented as mean ± SD (*n* = 5).

Next, we used MTT assay to detect the cell survival rate after treatment with LA-PEI and LA-PEI-PBA for 48 h. Figure 8A shows that when the carrier concentration is 40 *μ*g/mL, the KMM cell survival rate is maintained at about 80% after incubation with LA-PEI and LA-PEI-PBA, but when the concentration is greater than 40 *μ*g/mL, the cytotoxicity is concentration-dependent, that is, when the concentration increases, the toxicity increases. Figure 8B shows that when the carrier concentration is 20 *μ*g/mL, the SK-RG cell survival rate of LA-PEI and LA-PEI-PBA is maintained at about 85%, however, when the concentration is greater than 20 *μ*g/mL, the cytotoxicity is also concentration-dependent. The optimal mass ratio of nanocarriers to miR-34a-5p was 5:1, and the amount of nanocarriers was only 10 *μ*g/mL, which would not cause toxicity. Therefore, the hemolysis test, stability test, and cytotoxicity test showed that the nanocarriers have good biosafety.

### 3.5 Delivery effect of drug-carrying nanocomplexes on miR-34a-5p

**FIGURE 9:**
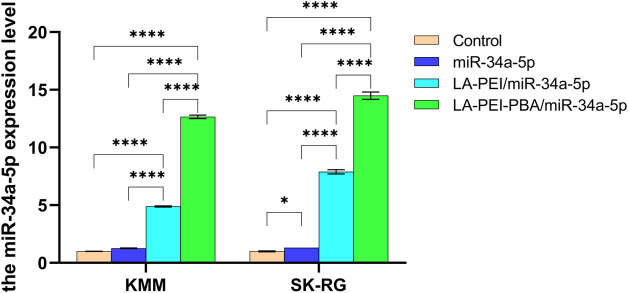
The level of miR-34a-5p in KMM and SK-RG cells. Data are presented as mean ± SD (*n* = 3), *p < 0.05, ***p < 0.001, ****p < 0.0001. (The mass ratio of carrier to miR-34a-5p was 5:1, and the miR-34a-5p was 2 *μ*g).

We further detected the level of miR-34a-5p in KMM and SK-RG cells by RT-PCR to verify the delivery effect of nanocarriers. As shown in Figure 9, compared with the control group, the level of miR-34a-5p in the free miR-34a-5p group did not significantly increase, indicating that the degradation of free miR-34a-5p had already occurred before it entered the cells. In the LA-PEI/miR-34a-5p group treated with KMM and SK-RG, the levels of miR-34a-5p were 4.89 and 7.89 folds, respectively. In the LA-PEI-PBA/miR-34a-5p group, the level of miR-34a-5p was 12.66 and 14.50 folds. miR-34a-5p in the LA-PEI-PBA/miR-34a-5p group was significantly higher than in the LA-PEI/miR-34a-5p group, indicating that PBA-modified nanocarriers are more conducive to cell uptake, which may be attributed to the targeting of PBA. These results also indicated that after the drug-carrying nanocomplex entered the cells, in the acidic environment of the endosome, the “proton sponge effect” of PEI increased the osmotic pressure of the endosome and caused the membrane to break down (Bus et al., 2018; Vermeule et al., 2018) so that the swallowed drug-carrying nanocomplex escaped and then released miR-34a-5p.

### 3.6 Cellular uptake

PBA ligands modified on drug-carrying nanocomplexes can also bind specifically to overexpressed sialic acid (SA) receptors on tumor cell membranes, thus targeting KSHV-infected cells. To facilitate the observation of the uptake of drug-carrying nanocomplexes, we used miR-34a-5p with red fluorescent dye Cy3 for fluorescence microscopy. KMM cells were treated with free miR-34a-5p-Cy3, LA-PEI/miR-34a-5p-Cy3, and LA-PEI -PBA/miR-34a-5p-Cy3 groups, and the results are shown in Supplementary Figure S1. The red fluorescence of the free miR-34a-5p-Cy3 group was not visible in the cells, and the LA-PEI/miR-34a-5p-Cy3 group showed little red fluorescence, while the LA-PEI-PBA/miR-34a-5p-Cy3 group showed more fluorescence intensity, indicating that PBA-modified carriers were more conducive to delivering miR-34a-5p into cells. This indicates that PBA-modified drug-carrying nanocomplexes have excellent targeting ability, which is conducive to the enrichment of miR-34a-5p in the cell.

### 3.7 Effects of drug-carrying nanocomplex on cell proliferation

**FIGURE 10:**
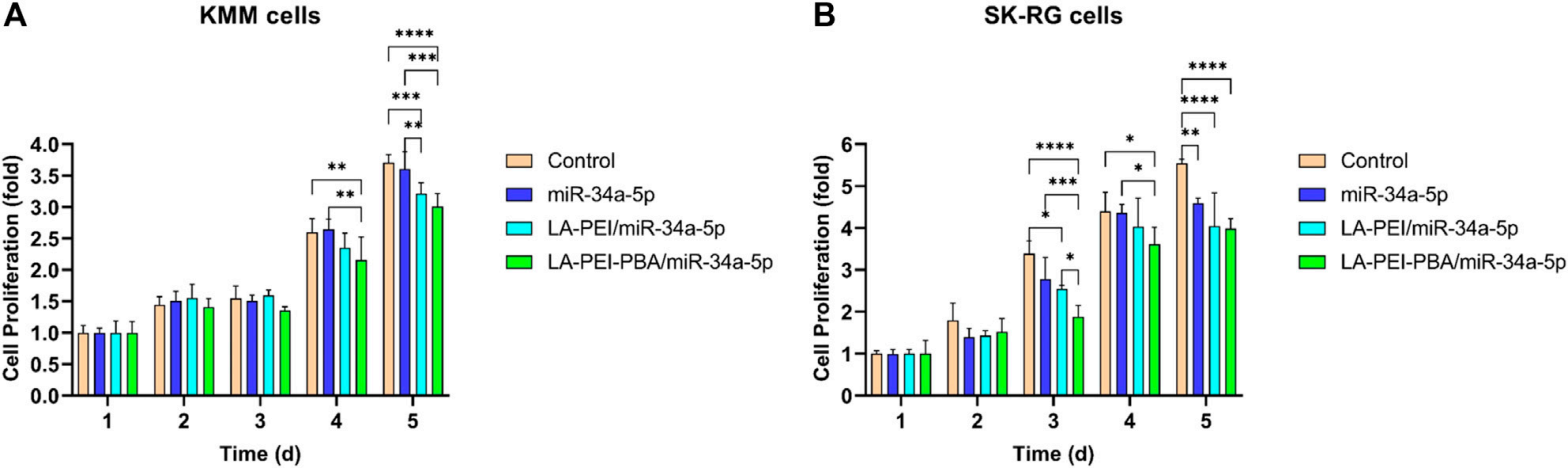
Cell proliferation. **(A)** Effect of drug-carrying nanocomplexes on proliferation of KMM cells and **(B)** SK-RG cells. Data are presented as mean ± SD (*n* = 5), *p < 0.05, **p < 0.01, ***p < 0.001, and ****p < 0.0001.

The effects of LA-PEI/miR-34a-5p and LA-PEI-PBA/miR-34a-5p on cell proliferation were detected by MTT assay. Figure 10A shows that KMM cell proliferation was not inhibited in all experimental groups at 1–3 days. From the 4th day, both the LA-PEI/miR-34a-5p and LA-PEI-PBA/miR-34a-5p groups could inhibit cell proliferation, and the inhibitory effect was more obvious in the LA-PEI-PBA/miR-34a-5p group. As shown in Figure 10B, the LA-PEI/miR-34a-5p group and LA-PEI-PBA/miR-34a-5p group showed inhibitory effects from day 3, and the LA-PEI-PBA/miR-34a-5p group showed stronger inhibitory effects. In conclusion, drug-carrying nanocomplexes have a better inhibitory effect on cell proliferation, and PBA-modified drug-carrying nanocomplexes are more conducive to inhibiting cell proliferation.

### 3.8 Effects of drug-carrying nanocomplex on cell migration

**FIGURE 11:**
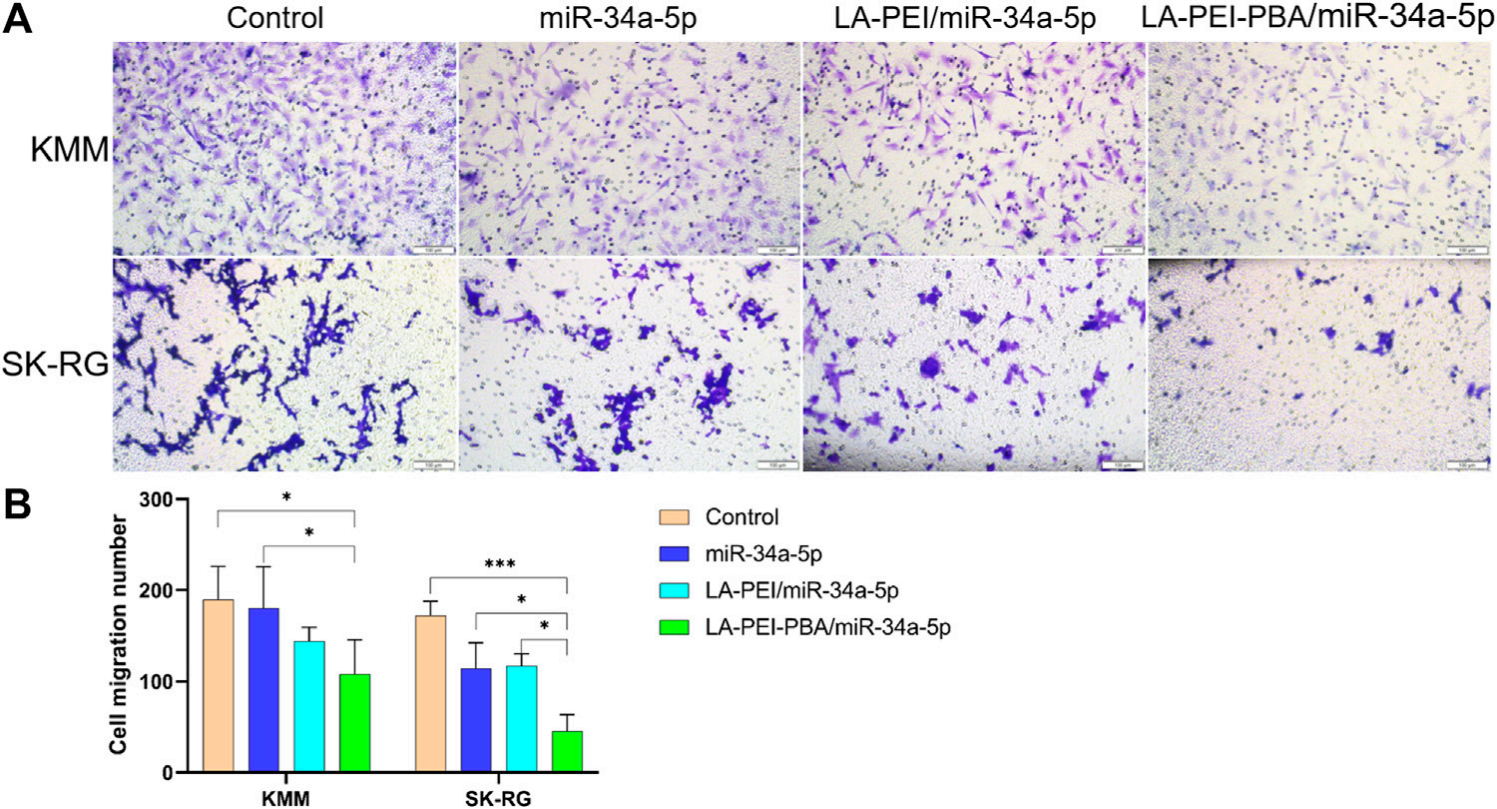
Cell migration. **(A)** Cell migration microscopy of KMM and SK-RG cells treated with drug-carrying nanocomplexes (scale: 100 μm), and **(B)** cell migration number. (The mass ratio of carrier to miR-34a-5p was 5:1, and the miR-34a-5p was 2 *μ*g). Data are presented as mean ± SD (*n* = 3), *p < 0.05, ***p < 0.001.

Cell migration plays an important role in the process of tumor cell metastasis, so inhibiting migration is also one of the methods of tumor treatment. As shown in Figures 11A, B, the number of cell migrations in the free miR-34a-5p group was similar to the control group, and the number of cells after treatment in the LA-PEI/miR-34a-5p and LA-PEI-PBA/miR-34a-5p group was significantly reduced. LA-PEI-PBA/miR-34a-5p group had a stronger inhibitory effect on cell migration. In SK-RG cells, the number of cell migrations of the free miR-34a-5p group was less than that of the control group, and the LA-PEI/miR-34a-5p group was very similar to it, while the LA-PEI-PBA/miR-34a-5p group showed a significant inhibitory effect, which may also be caused by the targeting effect of PBA. These results indicate that LA-PEI-PBA/miR-34a-5p could inhibit cell migration.

### 3.9 Expression of KSHV-related genes

**FIGURE 12:**
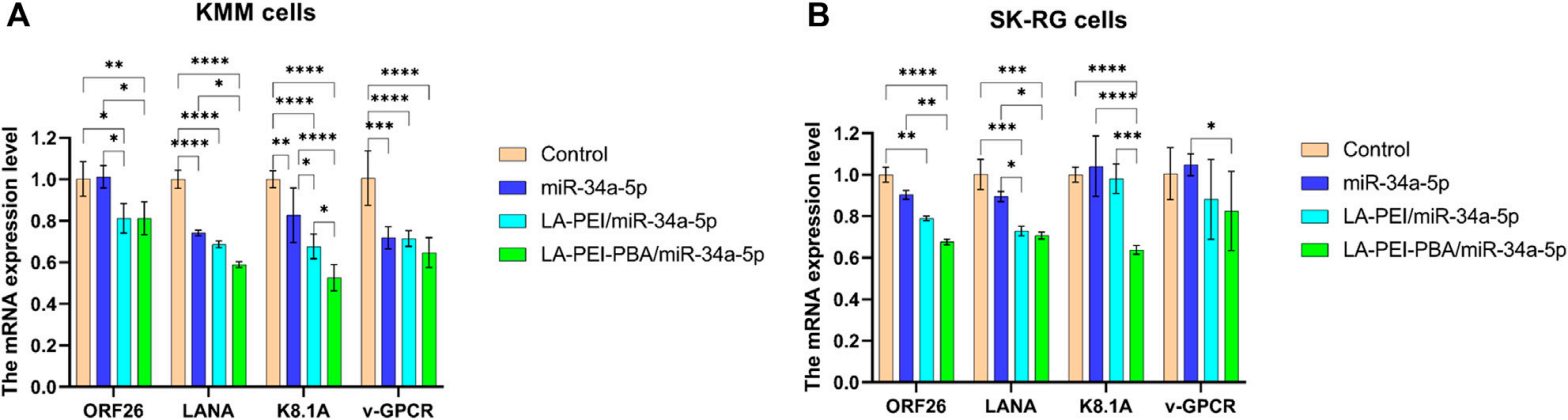
Effect of drug-carrying nanocomplexes on the expression level of KSHV-related genes. **(A)** KMM cells, **(B)** SK-RG cells. (The mass ratio of carrier to miR-34a-5p was 5:1, and the miR-34a-5p was 2 *μ*g). Data are presented as mean ± SD (*n* = 3), *p < 0.05, **p < 0.01, ***p < 0.001, and ****p < 0.0001.

After KSHV infections, there are two states: the latent and lytic state (Juillard et al., 2016). We further used RT-PCR to detect the expressions of the KSHV latent gene LANA and lytic genes ORF26, v-GPCR, and K8.1A to verify whether LA-PEI-PBA/miR-34a-5p could effectively suppress their expression. As shown in Figures 12A, B, LA-PEI-PBA/miR-34a-5p could effectively inhibit the expression of the KSHV latent gene LANA and the lytic genes ORF26, v-GPCR, and K8.1A in both KMM and SK-RG cells, proving that LA-PEI-PBA/miR-34a-5p has an anti-KSHV therapeutic effect.

## 4 Conclusion

In summary, in this study, LA-PEI-PBA, a cationic copolymer nanocarrier with a PBA targeting function, was successfully prepared through an amidation reaction. The drug-carrying nanocomplex LA-PEI-PBA/miR-34a-5p was formed by electrostatic adsorption of miR-34a-5p by nanocarriers. With the optimal mass binding ratio of 5:1, a particle size of 207.3 nm, and a potential of 26.74 ± 2.36 mV, it can effectively protect miR-34a-5p from nuclease degradation, and possess good stability, blood compatibility, and cell compatibility. A PBA-modified drug-carrying nanocomplex is conducive to cell uptake and increases the level of miR-34a-5p, thereby inhibiting the proliferation and migration of KSHV-infected cells, and reducing the expression levels of KSHV lytic and latent genes. The nano-drug delivery system constructed by this simple method may have a good application prospect in anti-KSHV treatment, and provide more strategies for the delivery of other nucleic acids.

## Data Availability

The original contributions presented in the study are included in the article/Supplementary Material, further inquiries can be directed to the corresponding authors.
