# Supplementary material for: Targeted delivery of miR-34a-5p by phenylborate-coupled polyethylenimide nanocarriers for anti-KSHV treatment
Source: Front Bioeng Biotechnol. 2024 Jan 8;11:1343956. doi: 10.3389/fbioe.2023.1343956 (PMC10801047; doi:10.3389/fbioe.2023.1343956)
Supplement: Supplementary file 1 [file DataSheet1.docx]

**Supplementary Material**

**Table S1. The sequences of primers**

| **Target Gene** | **Primer sequence** |
| --- | --- |
| miR-34a-5p | F: CGCGTGGCAGTGTCTTAGCT |
|  | R: AGTGCAGGGTCCGAGGTATT |
|  | RT:GTCGTATCCAGTGCAGGGTCCGAGGTATTCGCACTGGATACGACACAACC |
|  |  |
| U6 | F: AGAGAAGATTAGCATGGCCCCTG |
|  | R: ATCCAGTGCAGGGTCCGAGG |
|  | RT:GTCGTATCCAGTGCAGGGTCCGAGGTATTCGCACTGGATACGACAAAATA |
|  |  |
| ORF26 | F:CGAATCCAACGGATTTGACCTC |
|  | R:CCCATAAATGACACATTGGTGGTA |
|  |  |
| K8.1A | F: AAAGCGTCCAGGCCACCACAGA |
|  | R:CCCATAAATGACACATTGGTGGTA |
|  |  |
| LANA | F:AGCCACCGGTAAAGTAGGAC |
|  | R:GATGTGACCTTGGCGATGAC |
|  |  |
| v-GPCR | F: GTGCCTTACACGTGGAACGTT  R: GGTGACCAATCCATTTCCAAGA |
| β-actin | F: CGGAACCGCTCATTGCC |
|  | R: ACCCACATCGTGCCCATCTA |


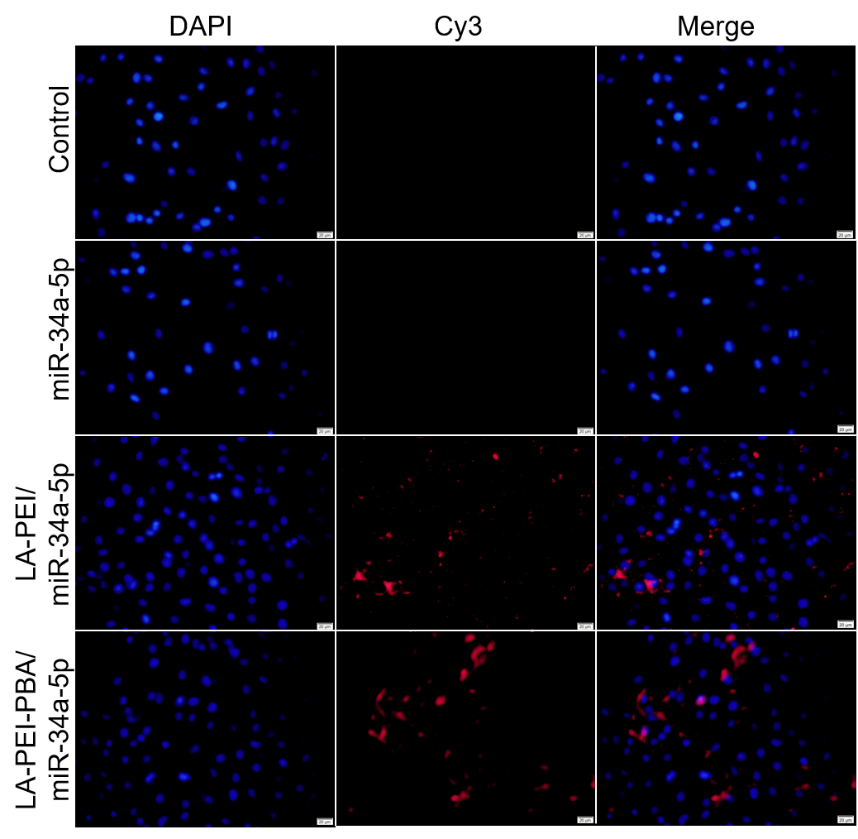


**Figure S1. Fluorescence microscope images of KMM cells treated with miR-34a-5p, LA-PEI/miR-34a-5p and LA-PEI-PBA/miR-34a-5p (scale: 200 μm).**

**
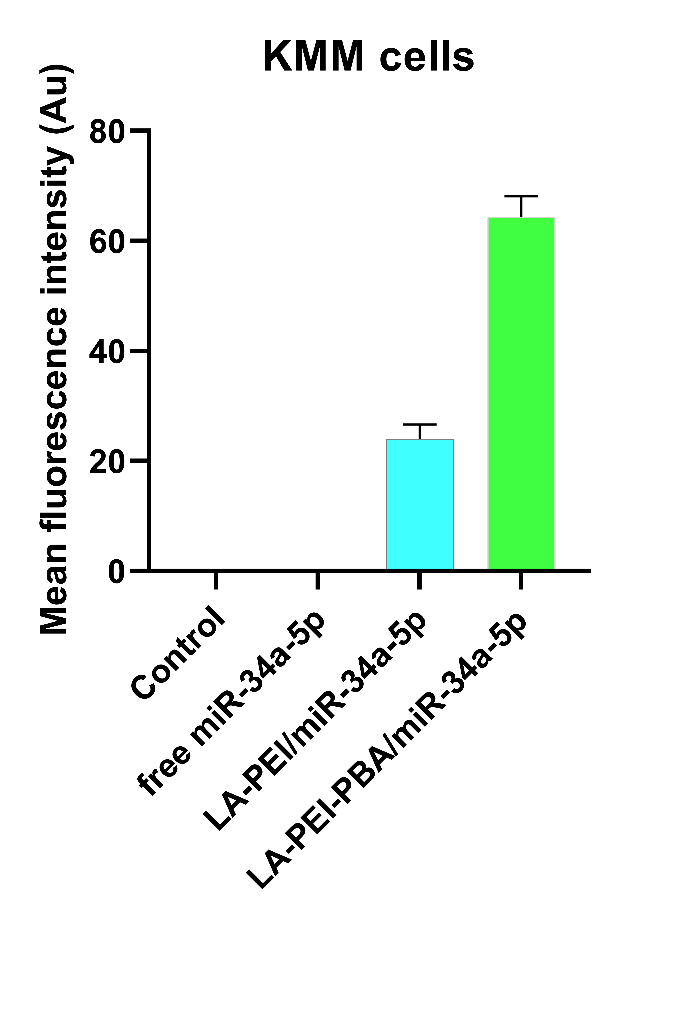
**

**Figure S1.** **Quantization of mean fluorescence intensity of drug-carrying nanocomplexes ingested by cells.**
